# Nutrient composition of fish protein powder developed from *Brycinus nurse* (Rüppell, 1832)

**DOI:** 10.1002/fsn3.844

**Published:** 2018-10-25

**Authors:** Nasser Kasozi, Denis Asizua, Gerald Iwe, Victoria Tibenda Namulawa

**Affiliations:** ^1^ Abi Zonal Agricultural Research & Development Institute National Agricultural Research Organisation Arua Uganda; ^2^ National Agricultural Research Organisation Entebbe Uganda

**Keywords:** *Brycinus nurse*, fish powder, Lake Albert, Pelagic fish

## Abstract

A new product in the form of edible fish powder was developed from small pelagic fish (*Brycinus nurse*) which constitutes one of the major commercial fisheries in Lake Albert, Uganda. The objective of the study was to determine the proximate and mineral composition of the newly developed fish powder and also to compare it with Nile tilapia powder already on market. Results indicated that the changes in the amount of protein and ash were found to be significantly higher (*p* < 0.05) in fish powder than in fried samples. The increase in fat content of fried fish samples was found to be significant compared to other fish samples. No significant changes (*p* > 0.05) were observed in carbohydrate content for raw, fried, and powdered samples. Vitamin A decreased significantly in powdered samples. Comparing the *B. nurse* powder and tilapia powder (made from juvenile tilapia as raw material), the results indicated that *B. nurse* had higher levels of iron and manganese. Extracting proteins from *B. nurse* provides an opportunity to increase the utilization of harvested catch.

## 1 INTRODUCTION

**Figure 1:**
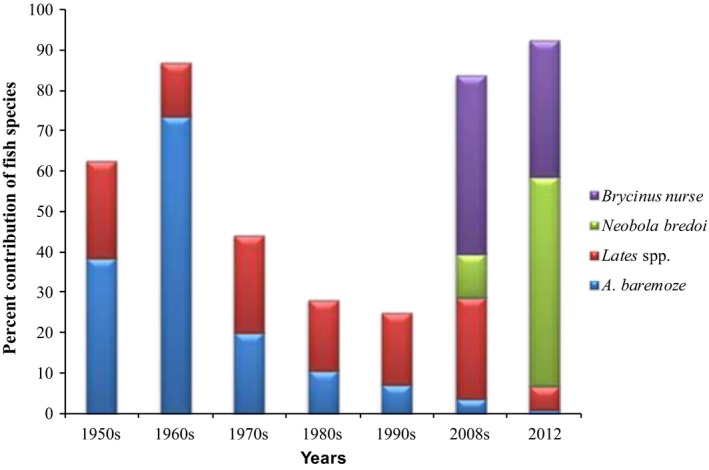
Percent contribution of *Brycinus nurse* landed on Lake Albert from 1950's. (Adapted from Mbabazi et al., 2012)

Nurse tetra*, Brycinus nurse* locally known as “Ragoge” or “Onangnanga,” is small‐sized pelagic, potamodromous species found mainly in African freshwater lakes (Azeroual & Moelants, 2010; Masette, 2013). It belongs to the family of Alestidae and is one of the highly demanded food fish species. The fish is endemic to Lake Albert, accounts for the largest volume (80%) of the total fish catch, and grows to a maximum size of almost 20‐cm fork length (Mbabazi et al., 2012; Masette, 2013). Catch statistics between the 1950s and 2008 indicate that *B. nurse* fishery is one of the emerging commercial fisheries on Lake Albert (Figure 1). Thus, production of fish protein from *B. nurse* is vital since the fish contributes substantially to the catches and there is availability of raw material at a reasonably cheap price. Shaviklo (2015) defined fish protein powder as dried and stable fish product, intended for human consumption, in which the protein is more concentrated than in the original fish flesh.

The Ugandan fisheries sector has been recognized as a key resource in tackling food and nutrition security issues and features prominently in the Development Strategy and Investment Plan (Kasozi, Rutaisire, Nandi, & Sundaray, 2017). Until recently, only large‐sized fish was exploited for human consumption while small‐sized pelagics were channeled to animal feed production systems (Kabahenda, Amega, Okalany, Husken, & Heck, 2011). In order to ensure that food supply optimally fulfills population nutrient requirements, utilization of small‐sized fish species could contribute considerably to reduce the level of micronutrient and protein malnutrition (Abbey, Glover‐Amengor, Atikpo, Atter, & Toppe, 2017).

In Uganda, *B. nurse* is generally consumed in fried form and is relatively cheap compared to other fish species such as Nile perch, pebbly, and tilapia. Despite the huge harvest of this fish, sun‐drying and deep‐frying have been the principal methods of preservation (Masette, 2013). Although these methods are affordable by the majority of processors, sun‐drying, for example, is weather dependent characterized by low processing capacity which implies that during the rainy season, the post‐harvest losses may be as high as 90% of the total catch. It also requires a large drying area, which in most cases is shared between different uses. The incomplete drying coupled with poor handling practices contributes substantially to the current high post‐harvest losses. Consequently, the high post‐harvest losses have severely undermined the utilization of this fish in Uganda, and thus, alternative processing techniques could be developed. Developing high‐quality fish products from *B. nurse* that can easily be stored and transported could contribute significantly to the reduction in protein malnutrition in Uganda. In addition, generating knowledge of the nutrient composition of *B. nurse* subjected to different processing approaches is vital in understanding the links between food production, access and nutrient intakes, and in devising policies and programmes such as the development of improved production technologies. Therefore, the aim of this study was to determine the nutritive value of raw and developed fish powder from *B. nurse* and to make comparisons with Nile tilapia powder already on market.

## 2 MATERIALS AND METHODS

### 2.1 Materials

A total of 300 freshly caught *B. nurse* was purchased from Abok landing site. Purchased fish samples were transported in insulated fish boxes to Kati Farms Uganda Limited. Fish samples were cleaned, descaled, degutted, and then divided into three groups (100 fish each). The fish samples were processed into fried products and powdered forms using improved artisanal methods. Samples were then coded and packaged in aluminum foil before storage in laboratory freezer, model UDD 500 BK made in Turkey at −10°C pending proximate and mineral analysis. All the prepared samples were then taken for analysis to Chemiphar (U) Ltd, an independent analytical laboratory, internationally accredited according to ISO 17025:2005.

### 2.2 Sample preparation

Fried samples were prepared as per the method described by Masette and Tinyiro (2016). After drip‐drying for 2 hr, the samples were fried in a locally fabricated mild steel shallow pan containing vegetable oil—Golden fry vegetable oil (fractionated palm oil, fully refined and fortified with vitamin A) made by Bidco (U) Ltd. The oil was heated to 180°C using gas cooker, and the frying time was 5 min. The excess oil was allowed to drain off the deep‐fried samples on a stainless steel mesh tray for 1 hr before packaging in aluminum foil and coding. Samples were then stored in a freezer at −10°C overnight (12 hr).

**Figure 2:**
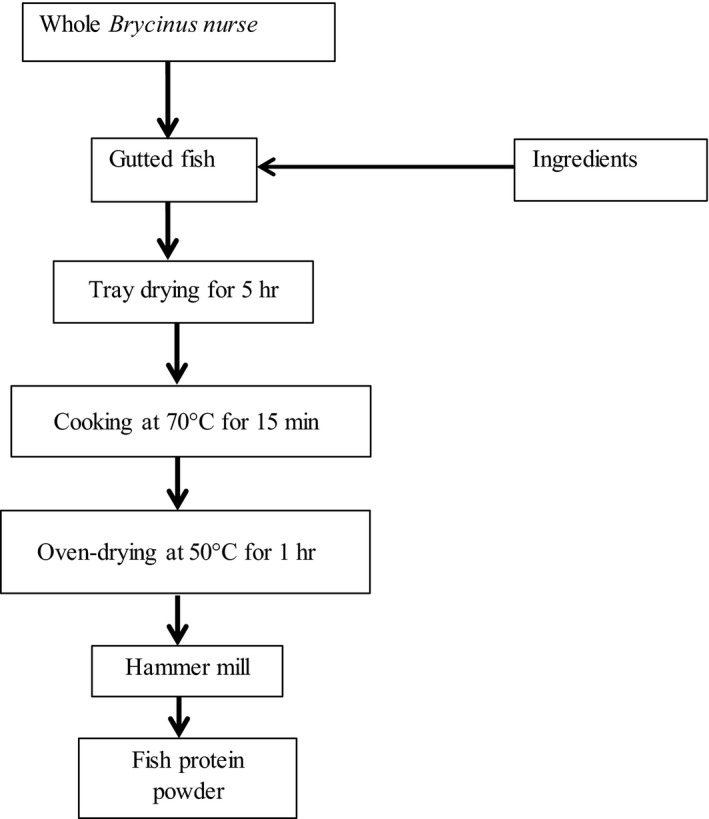
Flow chart for fish protein powder production

Edible fish powder was prepared as per the method previously described by Masette (2013) with some modifications. The process is shown as the flowchart (Figure 2). This method is effective in sustaining the high protein and original taste. Freshly caught *B. nurse* was washed with 1% brine and weighed. The ingredients that included garlic and onions were mixed thoroughly with fish before being heated at 70^°^C for 15 min and later oven‐dried at 50°C for approximately 1 hr. The resultant product was milled to produce a powder that was packaged in 200‐g quantities in readily available plastic containers and stored at room temperature.

### 2.3 Proximate analysis

For proximate composition, moisture content was determined by oven‐drying at 105°C to constant weight following the procedure of AOAC (1995). The crude protein content was determined by Kjeldahl method. Crude fat was determined following the procedure of AOAC (1995) by semicontinuous solvent extraction method (Soxhlet method). Ash content was determined after combustion for 20 hr at 550°C (AOAC, 1990). Total carbohydrate was determined by subtracting the sum of fat content, protein content, ash content, and moisture from 100.

### 2.4 Vitamin A

Vitamin A was analyzed by high‐pressure liquid chromatography (HPLC), as described by Manz and Philipp (1981). Vitamin A was saponified using ethanolic potassium hydroxide (50%). Saponified samples were then extracted with 70 ml of diethyl ether, three times, in a separating funnel. The extracts were combined and washed with distilled water before being concentrated and made to volume with ether in a 250‐ml volumetric flask. The quantification of vitamin A was done by HPLC with the following conditions: detection: fluorescence detector; excitation: 325 nm; emission: 480 nm; mobile phase: 98% n‐hexane; 2% propan‐2‐ol.

### 2.5 Mineral analysis

The preparation of samples for mineral elements analysis followed a method described by AOAC (1995). Approximately 5 g of respective raw, fried, and powdered samples were placed in a Teflon digestion vessel and double acid digested with nitric acid (HNO_3_) and perchloric acid (HCLO_4_). Samples were then analyzed for mineral contents of iron (Fe), manganese (Mn), zinc (Zn), potassium (K), calcium (Ca) using the atomic absorption spectrophotometer (Shimadzu AAS, AA‐6300). Total phosphorus was determined by spectrophotometric vanadium phosphomolybdate method. The mineral concentration was expressed as mg mineral/kg fish dry weight.

**Table 1:** Proximate composition and vitamin A of raw and processed *Brycinus nurse*

	Raw	Fried	Powder
Moisture (%)	71.6 ± 0.49^a^	16.6 ± 0.21^b^	7.30 ± 0.14^c^
Crude protein (%)	17.5 ± 0.42^c^	39.1 ± 0.07^b^	50.4 ± 0.56^a^
Fat (%)	8.1 ± 0.14^c^	35.7 ± 0.07^a^	30.1 ± 0.07^b^
Ash (%)	2.3 ± 0.42^c^	8.4 ± 0.63^b^	12.3 ± 0.14^a^
Crude fiber (%)	0.3 ± 0.07^a^	0.2 ± 0.03^b^	0.1 ± 0.06^c^
Carbohydrates (%)	<0.1	<0.1	<0.1
Energy (kcal/100 g)	140.6 ± 0.21^c^	477.8 ± 0.07^a^	470.9 ± 0.49^b^
Vitamin A (mg/kg)	5.3 ± 0.014^a^	3.6 ± 0.03^b^	<0.06

Note

Results are means ± standard deviation of duplicates. Values with the different letters in the same row are significantly different (*p* < 0.05).

### 2.6 Statistical analysis

Statistical analyses were performed using GenStat 14th edition (64bit): VSN International Ltd. To test the differences between processing methods, one‐way ANOVA was performed. Treatment means were separated by Fisher's protected t test least significant difference (LSD) at the 5% level of significance.

## 3 RESULTS AND DISCUSSION

### 3.1 Proximate composition

All proximate composition of the raw and processed *B. nurse* is presented in Table 1. The proximate composition of raw *B. nurse* is similar to earlier reports on pelagic species marketed in the Lake Victoria region (Kabahenda et al., 2011). Due to resource limitations, only vitamin A was analyzed. Changes in moisture, protein, fat, fiber, and energy contents were found to be significantly influenced (*p *< 0.05) by the processing methods. In this study, the moisture content of the fish decreased significantly during the frying and fish powder production. The greatest water loss was found in powdered samples. Water loss, occurring during the production of fish powder, resulted in higher protein content. Similarly, after cooking, a significant water loss was reported by Weber, Bochi, Ribeiro, Victorio, and Emanuelli (2008) in silver catfish; Rosa, Bandarra, and Nunes (2007) and Ersoy and Ozeren (2009) in African catfish.

**Table 2:** Main and trace elements contents of *Brycinus nurse* powder (mg/kg DM)

	Raw	Fried	Powder
Ca	10,554.3 ± 0.49^b^	17,766.7 ± 1.83^c^	35,100.2 ± 0.35^a^
P	13,270 ± 28.28^b^	2,671 ± 1.41^c^	20,552 ± 3.53^a^
K	2,252 ± 3.53^c^	7,475 ± 35.9^a^	7,100 ± 0.14^b^
Fe	11.7 ± 0.35^c^	34.1 ± 0.07^b^	125.7 ± 0.28^a^
Mn	6.9 ± 0.07^c^	12.1 ± 0.21^b^	13.2 ± 0.35^a^
Zn	12.8 ± 0.28^c^	41.8 ± 0.42^b^	75.7 ± 0.14^a^

Note

Results are means ± standard deviation of duplicates. Values with the different letters in the same row are significantly different (*p* < 0.05).

The concentration of protein was highest in powdered samples (50.4%) followed by 39.1% in fried samples. The level of protein in the powdered fish sample is comparable to the levels in *Rastrineobola argentea* (*mukene)*. Kabahenda et al. (2011) assessed micronutrient and protein levels in low‐value fish and processing by‐products in fish from Lake Victoria region and found crude protein in the range of 53.0%–58.8% in *mukene*, a low‐value fish.

Fried *B. nurse* had a higher level of fat than either raw or powdered product, mainly due to the absorption of fat by the fish during the frying process. The highest increase in the fat content (35.7%) during frying was observed. Saguy and Dana (2003) reported that the increase in fat content can be attributed to the oil penetration on the fish fillet after water is partially lost by evaporation during cooking. In addition, similar results have been reported for African catfish (Rosa et al., 2007) and rainbow trout (Gokoglu, Yerlikaya, & Cengiz, 2004) fried in vegetable oils.

The total energy content varied greatly with a range of 140–470 kcal/100 g and is believed to be related to the variation in fat content. The relative ash content in powdered samples is likely related to the inclusion of bones, viscera, fins as edible parts during the production. Bogard et al. (2015) reported that inclusion of bones as edible parts during processing could lead to a higher ash content.

The vitamin A content of the powdered fish samples decreased significantly (*p* < 0.05) when compared to raw and fried samples. Vitamin A is believed to be less heat‐labile than the water‐soluble vitamins, and as a result, it is likely to be susceptible to destruction at high temperatures in the presence of oxygen (Ersoy & Ozeren, 2009).

### 3.2 Mineral content

Table 2 gives the mineral content (main and trace elements) in the raw, fried, and powdered products of *B. nurse*. The levels of calcium, phosphorous, and potassium were significantly higher in the powdered form than in raw or fried forms. Among the main elements, the most abundant were Ca and P in the powdered fish. The concentrations of Fe in raw and processed fish samples varied significantly with the highest mean concentration observed in powdered form while the lowest mean Fe concentration was observed in raw fish.

The Ca content of raw fish was found to be 10,554.3 mg/kg. The highest Ca content (35,100.2 mg/kg) was observed in powdered fish followed by (17,766.7 mg/kg) in fried fish. This value is higher than what reported by Ersoy and Ozeren (2009) in African catfish and Gokoglu et al. (2004) in rainbow trout but similar to that reported by Kabahenda et al. (2011) in *mukene* products within the range of 1,556.4–1,866.5 mg/100 g which is an indication that small pelagic fish species are rich in calcium compared to large‐sized fish species. As would be expected, calcium content is higher in species in which bones are commonly consumed and included in the edible parts.

The P content of raw fish was found to be 13,270 mg/kg. Decreased P content was noticed in fried fish. The increase in P content in powdered samples was found to be significant (*p* < 0.05). Decreased P content was reported in fried fish fillets of rainbow trout when compared to raw fish (Gokoglu et al., 2004).

The K content of raw fish was found to be 2,252 mg/kg. This result is similar to K contents of trout described by Wheaton and Lawson (1985) (2,800–3,580 mg/kg). However, the K contents of fried and powdered samples increased significantly (*p *< 0.05).

The Mn content of raw, fried, and powdered fish samples ranged from 6.9 to 13.2 mg/kg. The increase in Mn content after frying was found to be significant (*p* < 0.05). Similarly, Rosa et al. (2007) reported an increase in the Mn content of fried African catfish.

The Fe content of raw *B. nurse* was 11.7 mg/kg while for the powdered samples was 125.7 mg/kg. A similar finding was reported by Kabahenda et al. (2011) indicating significantly increased Fe in *mukene,* pelagic fish species. The change in Fe content after processing was found to be significant (*p *< 0.05). Recommended dietary intake (RDI) of iron for children 9–13 years/day is 8 mg (Abbey et al., 2017). Hence, consumption of *B. nurse* powder could adequately meet the iron needs of children in these age brackets.

The mean Zn content of *B. nurse* was found to be 12.8 mg/kg. The increase in Zn content after frying and powder production was found to be significant (*p* < 0.05). Wheaton and Lawson (1985) have reported that Zn content of trout ranged from 1.4 to 26 mg/kg. A similar observation was reported by Kabahenda et al. (2011) indicating significantly increased Zn in *mukene* products.

### 3.3 Comparison of *Brycinus nurse* and tilapia powder

The chemical composition of a protein powder from *B. nurse* and juvenile tilapia is represented in Table 3. This part of the study compared the nutritive value of the developed powder from *B. nurse* with protein powder already on market made from juvenile Nile tilapia (in the range of 10–20 g). There was no significant difference observed in moisture content among the two powdered samples (*p* > 0.05). Significantly higher protein content (66.7%) was recorded in tilapia powder than *B. nurse* powder (50.4%). Fish protein powder is influenced by raw material, production method, and different processing conditions (Shaviklo, 2015). Although tilapia powder had significantly higher content of protein and Ca, P, K, and Zn than *B. nurse*, acquiring suitable raw material of tilapia within the desired size of 10–20 g where all the bones and fins are included is a challenge. This size is highly demanded for pond and cage aquaculture as source of seed, and therefore, utilizing it for protein powder may be costly. Since mature *B. nurse* is typically consumed whole with head, bones, and in some cases, viscera, this product can be good substitute to fish powder made from juvenile Nile tilapia.

**Table 3:** Comparison of proximate and mineral analysis of developed *Brycinus nurse* powder with tilapia fish powder

	*B. nurse* powder	Tilapia powder
Moisture (%)	7.3 ± 0.14^a^	7.2 ± 0.07^a^
Crude protein (%)	50.4 ± 0.56^b^	66.1 ± 0.21^a^
Fat (%)	30.1 ± 0.07^a^	9.1 ± 0.07^b^
Ash (%)	12.3 ± 0.14^b^	17.2 ± 0.23^a^
Crude fiber (%)	0.1 ± 0.0^a^	0.1 ± 0.0^a^
Carbohydrates (%)	<0.1	<0.1
Energy (kcal/100 g)	470.5 ± 0.49^a^	347.6 ± 0.56^b^
Ca (mg/kg)	35,100.2 ± 0.35^b^	52,085 ± 7.07^a^
P (mg/kg)	20,552 ± 3.53^b^	30,554 ± 6.36^a^
K(mg/kg)	7,100.4 ± 0.14^b^	9,450 ± 0.56^a^
Fe (mg/kg)	125.7 ± 0.28^a^	15.1 ± 0.07^b^
Mn (mg/kg)	13.2 ± 0.35^a^	12.3 ± 0.07^b^
Zn (mg/kg)	75.7 ± 0.14^b^	217.2 ± 0.28^a^

Note

Results are means ± standard deviation of duplicates. Values with the different letters in the same row are significantly different (*p* < 0.05).

## 4 CONCLUSION

Funding and interventions have largely focused on capture fisheries, particularly of large carps and introduced species on Lake Victoria neglecting small pelagics on Lake Albert. The data presented here indicate that *B. nurse* holds the potential to provide a much greater contribution to food and nutrition security. The protein in the developed fish powder is more concentrated than in the original fish flesh and can be fit for human consumption. Thus, this fish protein powder could therefore serve as a good source of nutrients for the poor particularly in rural and urban areas where limited economic resources prevent dietary diversity. This study indicates opportunities to produce value‐added products from *B. nurse,* one of the low‐value/underutilized fish species.

## CONFLICT OF INTEREST

The authors declare that there is no conflict of interest regarding the publication of this paper.

## ETHICAL STATEMENT

This study does not include any animal or human testing or questionnaire analysis on market survey.
